# The International Homœopathic Convention

**Published:** 1881-09

**Authors:** 


### HOMŒOPATHIC PHYSICIAN.

A MONTHLY JOURNAL OF MEDICAL SCIENCE.

"If our school ever gives up the strict inductive method of Hahnemann, we are lost, and deserve to be mentioned only as a caricature, in the history of medicine."—CONSTANTINE HERING.

Vol. I. SEPTEMBER, 1881. No 9.

### THE INTERNATIONAL HOMŒOPATHIC CONVENTION.

THE following report of the Homœopathic Convention, held in London, July 11th–18th, is condensed from the British journals, the *World* and the *Review*. As both of these journals speak enthusiastically of it, we may conclude that their accounts of its proceedings are fair and unbiassed. The *H. World* says: "Rarely has it fallen to our lot to chronicle such a complete success." The *M. H. Review* says: "This important meeting, which has been anticipated for so long by many of us, the preparations for which have occupied so much of the time and thought of some, is now an event of the past. Happily, the retrospect it affords is one of undiluted, of unalloyed pleasure. * * * From a scientific point of view especially, the meeting was a success of a high order." Whether or not these tributes are merited, our readers can judge from the following extracts of the Convention's *work*.

The business part of the Convention commenced its sessions Monday, July 11th, by the address of Dr. Hughes, the President. After a touching reference to the life and character of the late Carroll Dunham, he also noticed the deaths of Drs. Quinn, Nüñez, Hering, Hempel, Grauvogl and Jahr, all occurring in the last five years. He then described the arrangements which had been made for securing papers and for facilitating discussion, and passed to the consideration of the objects aimed at in holding these meetings. These, he said, were—

*First.* The consideration of the best plans for propagating the method of Hahnemann. He urged that homœopathy was a method, and not a doctrine or system. Hahnemann had his theories, pathological, such as psora; physiological, such as dynamization; but there was no such thing as homœopathic pathology, no such thing as homœopathic physiology. He then considered the leading features of homœopathy—the principle, the dose, the single medicine—describing these as, collectively, the method bequeathed us by Hahnemann. He then vindicated the liberty of the physician who practiced homœopathy in the use of such measures as appeared to him to be best adapted to the individual case before him; arguing, at the same time, that departure from homœopathic prescribing was a grave responsibility—a responsibility that ought to be assumed only after a full conviction of its necessity.

*Secondly,* The Convention had in view the development of homœopathy.

*Thirdly,* The Convention would, it was hoped, have a powerful influence in cementing in friendly union the physicians practicing homœopathy in various parts of the world.

Dr. Pope was elected Vice-President; Drs. Talbot, Boston; Breyfogle, Louisville; Meyhoffer, Nice; and Drysdale, Liverpool, were elected Honorary Vice-Presidents. Then followed reports on the history of homœopathy in different parts of the world during the last five years; reports being presented by Dr. Martiny, for Belgium; by Drs. Logan and Nichol, for Canada; by Dr. Allan M. King, for the provinces of New Brunswick and Nova Scotia; by Dr. Claude, for France; in absence of Dr. Goullon, Jr., Dr. Dudgeon reported for Germany; Dr. Pope reported for Great Britain and the Colonies; Dr. Sircar, of Calcutta, reported the progress of homœopathy in India; Dr. Bernard Arnulphy reported for Italy. Dr. Bojanus, of St. Petersburg, opened his interesting account of homœopathy in Russia, with a notice of the report made to the late Emperor by military medical officers. The number of homœopathic physicians in Russia is about 200. Scant literature. Dr. Lloyd Tuckey spoke for Spain. The chief event in the last five years was the opening of the homœopathic hospital in Madrid. The Hahnemannian Society is very prosperous, and the journal *El Criterio Medico* has been enlarged. For the United States, the *diffident* Dr. Talbot reported 6,000 physicians [to whom only 1,000 copies of HAHNEMANN'S ORGANON have been sold!—Ed.], 26 organized State societies, over 100 local societies, 38 hospitals, 40 dispensaries, 11 medical colleges, and 17 journals.

After these reports had been made, a discussion ensued on "the condition and prospects of homœopathy at the present time, and the best means of furthering its cause." This problem was solved by Drs. Talbot, Claude, Dudgeon, De Gersdorff, Bushrod James, Pope, Leon Simon and others.

One gentleman—well known as an eclectic—exclaimed: "Give me (!) the *young* men to instruct, and I will guarantee the future of homœopathy." Can Punch or Puck beat *that?* Suppose Catiline had exclaimed: "Give *me* the *young* men of Rome to teach, and I will guarantee her future!"

The following subjects were discussed. We can only give abstracts. (From the *Monthly H. Review.*)

### *Thoughts on the Scientific Application of the Principles of Homœopathy in Practice.*

THOMAS HAYLE, M. D., Edin., of Rochdale.

Dr. Hayle commenced his paper by dwelling upon the importance of facts as distinguished from speculations, arguing that it was from rash speculations and reckless experiments that much of the evil that had resulted from the use of drugs in the past had accrued. Referring to the effect produced on Hahnemann by his reflections on the practice of medicine, and his resolution not to terminate his train of thought until he had arrived at a definite conclusion, he describes it as "a frame of mind of which it may be asserted, as an everlasting truth, that those who seek shall find, and that unto them who knock it shall be opened."

Briefly noticing the circumstances which led Hahnemann to the assertion of the law of similars as the basis of drug selection, to the researches made by him confirming its truth, and to such as have since been made, he points to them as having established Hahnemann's discovery beyond question.

Noticing Hahnemann's sole reliance upon symptoms and their most minute surroundings, with the result of setting them forth in a schema which was artificial, he proceeded to consider, from an historical point of view, the infinitesimal dose, describing it as a discovery as brilliant as any in the annals of medicine, and one to which the law was a step. Of the reception of homœopathy among its adherents, he said, the great majority materialized its teachings; their habits and instincts led them to compromise—they preferred the lower attenuations, often giving the crude material. Another branch of homœopathists out-Hahnemanned Hahnemann—he gave thirtieths, they gave millionths. He observed positions, aspects and the weather, and they attended to the most minute particulars and circumstances. That which Hahnemann did from necessity, they do from choice. The resources of pathology were not open to him, and he was therefore compelled to find his similar in a very roundabout way. Symptom covering was his only resource.

Encumbered as it has been, the achievements of homœopathy have been great; but what may not be expected when science has cleared away the impediments, and has revealed the essentials in their unadulterated beauty, when we shall have ascertained the nature, extent and limits of the law, and the essence and relative importance of the symptoms!

Dr. Hayle then passed to a consideration of a rational theory of medicinal action. * * *

Dr. Hayle then detailed a case where fever and pleuritic stitches were the result of exposure to a north-east wind, which was completely checked by one dose of *Aconite* 30. The next day the patient was free from pain and fever, but weak. In explaining the mode of cure in this case, he says: "Medicinal action consists in a particular mode of motion, controlling and altering the mode of motion which is constantly going on in the different nerves. It does not alter the mode of motion that is going on, if healthy, that is synchronous with its own mode of motion; but whatever is amiss, out of gear, it restores to its normal action, and, in fact, sets all right that is wrong." A large dose or low dilution not only acts on the diseased parts, but sets up morbid movements of its own, deranging the whole nervous tracts.

Comparing Stanley's account of his successful treatment of his marsh fever in Africa by large doses of *Quinine* with those recorded in Rückert's *Klinische Erfahrungen*, where small doses were used, Dr. Hayle says that he believes the cures wrought by the larger doses are more violent and less rapid, and more apt to return than those by smaller doses, which are accompanied with less struggle, as only the diseased parts are touched, while the healthy parts remain unaffected. In the smaller dose the vibrations are synchronous with the healthy parts, and only those which are out of gear are touched. In the other case the whole sphere of the medicine, that is, the sphere on which it acts, is abnormally and violently acted on.

In chronic cases, the vessels of the part are chronically dilated, and have lost their elasticity. Speedy relapse follows restoration by a single dose. This state of things is to be met by a skillful repetition of dose, and, if the part is accessible by a typical stimulant, or by large doses, we should not give a second dose until the first has exhausted its action, and we should persevere with our medicine as long as it seems to do good. Alternations impede the action of the right medicine, and prevent the acquisition of experience. "The charioteer in the car of homœopathy," says Dr. Hayle, "always drives at least a pair of horses, but rarely well matched." * * *

Dr. Hayle concluded by advocating the remodelling of the materia medica, by arranging the symptoms in the order of their occurrence. The doses in which the drugs have produced them should be stated, and the effects of a change of dose upon the nature and order of symptoms should be ascertained. The causes, seat and nature of the symptoms should be analyzed.

To accomplish this end, Dr. Hayle proposes the formation of an experimental committee. By such work all attempts to include truth by including everything, even the unimportant and minute, would be unnecessary. Transitional and temporary aberrations would be merged in one uniform and scientific system of practice, which might admit of additions but not of change.

---

### *Generalization and Individualization.**

R. Hughes, L.R.C.P., Edin., of Brighton.

In opening his paper, Dr. Hughes spoke of the necessity of defining the word "likes." In doing so, he described two classes of homœopathic practitioners, the one satisfied only when he can secure a drug which will produce the morbid state supposed to constitute the disease he is called upon to treat; while the other ignores disease for therapeutic purposes as a pathological state, and regards only sick persons. The totality of the symptoms is the sole guide to the simillimum, and if that is not attainable, reliance must be placed on the more peculiar symptoms. Dr. Hughes then proceeded to show, by quotations from *The Organon* and Hahnemann's *Lesser*

* Dr. Hughes's essay was well handled by Dr. Drysdale, who pointed out that generalization stood for pathology. To this Dr. Hughes was understood to assent.—*Hom. World.*

*Writings*, that, while Hahnemann taught that for the multitudinous and diverse forms of disorder which come before the physician, arising from common causes (atmospheric and such like), and having no permanent character, selection by totality of symptoms and treatment as individual maladies formed the best mode of proceeding, yet he ever recognized that there were a certain number of diseases of fixed type, acquiring this by origination from a specific (generally miasmatic) cause. To these he appropriated one or more specific remedies, as always applicable and usually indispensable. And, further, he considered it a positive gain when morbid states, hitherto regarded as individuals, could be referred to a common type and treated by remedies chosen from a definite group, instead of being made the subjects of an indiscriminate search through the materia medica.

From the evidence he adduced, showing that Hahnemann recognized certain specific forms of disease, which are always essentially the same, and always curable by the same remedy; that he divided miasmatic diseases into acute and chronic, and defined another class of diseases as specific fevers, each epidemic having features of its own, but all cases of each being amenable to the same specific remedy; that he asserted the value of the same remedy for the few diseases which have a constant character; and from the importance he attached to the facility afforded in prescribing by the recognition of the psoric origin of chronic disease; as well as from the fact that he acknowledged the curative power of *Spongia* over goitre; of *bark* in endemic malarial fever; of *Veratrum album* in the water colic of Lauenburg; of *Aurum* in suicidal melancholia; of the prophylactic power of *Belladonna* over scarlatina, and of *copper* over cholera. Dr. Hughes argued that Hahnemann was no mere individualizer, that he resorted to this method only where other guidance failed him, that for him there were morbid species and specific medicines, and that he counted it real gain to reclaim forms of disease from the desert of symptomatology, to trace them to a common origin and connect them with certain remedies.

Having thus shown that pure individualizers were without authority, he argued that they had no foundation in reason. To obtain a group of allied remedies, generic and specific characters are necessary. Generalization must precede individualization. Further, by generalization we are able to utilize the experience of the past.

There are cases, Dr. Hughes urged, such as goitre and mumps, where we must all generalize exclusively; others, such as nervous disorders, varieties of dyspepsia, and of defective nutrition, which cannot be conformed to any known type of disease, and here individualization is the only reasonable course. Between these two extreme poles there is an extensive zone of genuine morbid species, each requiring the allotment of a group of specific remedies to be differentiated in accordance with each variety and each case. Where, on the other hand, this is not possible, where the practitioner has to choose between a remedy producing symptoms similar to some of the peculiarities of the instance before him or to the type of disease of which the instance in question is a specimen, Dr. Hughes argued that it was of greater consequence to secure similarity to the pathological process itself than, to use Hahnemann's own words, "to some accidental concomitant circumstances which do not alter its essential character."

---

### *A New Similia.*

A. W. Woodward, M.D., Chicago.

Dr. Woodward defined disease as a combined picture of pathological lesion *plus* the special sympathetic disturbances attending it; necessitating a remedy, which is a simillimum, not only to the local lesion, but to all the symptoms in the order of their relative importance. Our drug provings fail, he said, in giving the combination and subordination of the symptoms peculiar to and characteristic of each drug, rendering us unable to estimate correctly the attending symptoms which govern the success of the remedy. A drug can only be radically curative when it presents a complete parallel to the totality of the disease symptoms. If it cures to-day and fails to-morrow in the same disease, it must be owing to differences existing, not in the local lesion itself, but in the epiphenomena which modify and present a favorable result, and to which the drug is not homœopathic. To obtain the knowledge necessary for prescribing in this manner, Dr. Woodward argued that provings must be made on the healthy by a single dose taken in sufficient quantity to produce disturbance of the entire economy. Dr. Woodward then adduced a series of provings of *Arsenic, Nux vomica, Cinchona, Veratrum album, Aconite* and *Belladonna,* which were brought forward to show (1st) That the same drug when taken in health, and in a single dose, will affect many persons in the same general manner, though the special symptoms will vary; (2d) That all medicines begin their action by excitement, either of the motor, the sensory, or the excretory functions; and that they divide themselves naturally into three groups or classes, according to the order in which their general functions are disturbed successively; (3d) That each drug, while exhibiting the general method of action belonging to its class, shows its individuality by the succession in which it disturbs the special organs and functions of the body, thus presenting a combination of symptoms peculiar to that drug alone.

In the proving of *Arsenic* by three persons—two male and one female—the single dose was, in one case, three drops of the 1x, in a second, a grain and a half of the 1x trituration, and in the third, three grains of the 2x. An analysis of the provings showed that, while special symptoms varied, uniformity of physiological action was seen in the symptoms beginning with morbid sensations, and being followed by morbidly increased or altered secretions—with a final general disturbance of a febrile character. These provings are held to show that *Arsenic* disturbs not only special organs, but the entire economy in one specific direction, and that these disturbances are cumulative. Its use then, clinically, must be governed, not alone by the local symptoms of disease, for they may belong to many drugs, but by the associated sympathetic disorders that must always characterize this remedy in any disease. Thus, excluding the *locus morbi*, gastric symptoms always lead, cephalic are next in importance, and cutaneous, respiratory, spinal, renal and enteric each progressively decrease in importance, except when one of them becomes the leading feature as the seat of disease.

The new similia governing the use of *Arsenic* in disease is, that whatever the disease may be called, the indications for this drug are invariable, and will be limited to only two conditions. 1st. That the sufferings and morbid excretions shall exceed the fever. 2d. That the chief sympathetic disorder must always be gastric, the second cephalic, the third, cutaneous, etc. In this manner, Dr. Woodward examined the provings he had conducted of the medicines already named.

---

### *On the Alternation of Medicines.*

Dr. Martiny, of Brussels, and Dr. Bernard, of Mons, Belgium.

The authors define alternation as the successive administration of two or more remedies which recur in turn in a regular order and at intervals sufficiently approximated, so that the duration of the action of the one drug may not be quite exhausted before another succeeds it.

This methodical alternation they consider constitutes an important step in practical progress.

In taking a retrospective view of the practice of alternation, they refer to Hahnemann, who, in the edition of the *Organon* published in 1810, admitted its necessity, because of the "insufficient number of remedies tried up to that time."

Hering, Gross, Rummel, Ægidi, Kœmpfer, Hirsch, Hartmann and Perry are cited as supporting the alternation of medicines in the early history of homœopathy, and Teste, Jousset, Mouremans, Espanet and Van den Necker as doing so in later years.

The ideal of the practice of homœopathy, the finding of a remedy whose pathogenetic symptoms comprise the totality of the morbid symptoms, actual and antecedent, personal and hereditary, objective and subjective, is, they say, one bristling with difficulties—difficulties which have led to the alternation of drugs. They doubt whether the progress of therapeutics will ever bring us exclusively and definitely to the simplicity, so seductive, and, in appearance at least, so much more logical, of the administration of one single remedy; and consider that so long as this ideal or even unrealizable perfection of the method is not attained, it is, from a clinical point of view, advantageous in ordinary practice to habitually alternate remedies two by two, or three by three, or even four by four, when two or three drugs are not sufficient to cover all the symptoms, or do not answer to all the causes of disease, both profound and occasional. For example, an acute pleurisy occurs in an emphysematous patient who has had hæmorrhoidal troubles:—*Aconite* will be alternated with *Bryonia* and *Arsenic;* and when the acute symptoms are calmed, we believe that to obtain a prompt and durable cure, we must give *Bryonia* the first day, *Arsenic* the second, *Nux vom.* the third, and perhaps *Sulphur* the fourth.

They then illustrate this method of prescribing by reports of a series of cases, in each of which several remedies were used either in alternation or succession.

In discussing the *modus agendi* of medicines thus prescribed, they argue, 1st, that sometimes they act as adjuvants, and instance *Spongia* and *Hepar* in croup, and *Aconite* in acute inflammation, alternated with *Belladonna* or *Mercurius*, etc.

2d. They act sometimes as correctives—as in cases where special susceptibilities to the action of certain medicines exist—as when *Sulphur* cannot be taken singly; but when alternated with *Nux* it does good, while the *Nux vom.* alone would be inefficacious.

3d. They think that sometimes alternated remedies seem to constitute a new medicinal means endowed with new properties, illustrating this by Dr. Kafka's experience, who says that he has cured chronic catarrhs of the stomach by alternating *Nux vom.* and *Calcarea* after having uselessly administered these two remedies singly.

4th. That under the influence of remedies of more or less different, sometimes even antidotal action, the remedy seems to react more briskly; the vitality seems to emerge from the torpor into which it appeared plunged.

They next proceed to consider the objections made to alternation.

1st. Alternations were condemned by Hahnemann.

2d. With alternation it becomes difficult or impossible to discuss the characteristic effects of each of the agents employed. The object of giving remedies being to cure and not to experiment, they regard this objection as having no weight.

3d. The alternation of medicine is nothing more or less than a disguised return to polypharmacy. This objection they assert is only a specious one. Polypharmacy means the simultaneous employment or mixture in one formula of several different substances, whilst the method advocated consists in the employment of single remedies at short intervals.

4th. The alternation of medicines, if elevated to a system, will simplify too much the practice of homœopathy; it will favor the laziness of medical men, and the usurpation of the art by outsiders.

The simplification of the practice of homœopathy, so far from being matter for regret, should, they argue, be considered as a benefit.

5th. We can admit strictly the alternation of two medicines, but that is the extreme limit of the concession we can make to the partisans of alternation.

This objection they regard as specious, as, if it is admitted that two remedies may be alternated, there can be no valid reason why a greater number should not be used in succession.*

The President now resumed the chair, and a discussion on the Alternation of Remedies, opened by Dr. Clark, took place.

At its conclusion the following papers were presented:

---

* Excellent! Why not give the whole Mat. Med. in every case?

### *Drug Attenuation: Its Influence upon Drug Matter and Drug Power.*

JABEZ P. DAKE, M. A., M. D., Nashville.

Dr. Dake opened his paper by stating that the remedy to be employed in the combat with disease, upon whatever therapeutic principle or theory chosen, must be exhibited in proper form and quantity, to the end that its influence may be satisfactory. What, then, he asks, is the effect of drug attenuation upon drug matter? What its effect upon drug power?

Drug attenuation is defined as the diminution of a drug mass by division and subdivision and admixture with some neutral or non-medical substance as a menstruum or vehicle.

Viewing the question historically, he showed that Hahnemann adopted this method of dealing with drugs. 1st. To avoid aggravation of disease from too large a dose. 2d. To secure a thorough diffusion of drug particles. 3d. He claimed that through a better preparedness for absorption and an increased surface for contact increased power was obtained. 4th. A given dose of a homœopathic remedy was increased in power by the increased susceptibility to it produced by disease. 5th. In order to explain or account for the action of infinitesimals, Hahnemann broached the theory that medicine does not act atomically, but dynamically. 6th. Hahnemann conceived the idea that vigorous succussion and trituration effected a great unknown and undreamed of change by the development and liberation of the dynamic powers of the medicine.

Passing to the later history of drug attenuation, Dr. Dake described Korsakoff's "dry contact potencies," putting one dry medicated globule in a bottle full of pure sugar pellets in order to medicate the whole; Jenichen's high potencies; those of Lehrmann and Fincke—all of whom had, Dr. Dake observed, exceeded the utmost limits thought of by Hahnemann in the diminution of drug matter and development of drug power.

After noting the observations upon trituration of Segin and Mayhofer made with the microscope, those of Dr. Breyfogle made with chemical reagents, those of Professor Edwards Smith, Professor S. A. Jones, Dr. Lewis Sherman and Professor Conrad Wesselhœft with the microscope, those of Professor Wesselhœft with the spectroscope, and some of the teachings of analogy, which, Dr. Dake says, compel us to conclude that potent drug material may exist in attenuations, where every test save that of the living animal organism fails to detect its presence, he thence drew the inferences: 1st. That medicinal substances differ greatly in their cohesive property and divisibility. 2d. That some may be readily diffused in minute particles through a menstruum. 3d. That others are comminuted with great difficulty and slowly. 4th. That in the case of some metals the comminution is much more complete by chemical than by mechanical measures. 5th. That in the decimal or centesimal scale the theoretical or mathematical rate of diminution in the size of the particles is very different from the actual. 6th. That by chemical reagents drug matter can be recognized in no decimal attenuation above the third; by the spectroscope, in none above the seventh; and by the microscope, in none above the eleventh or twelfth. 7th. That analogy warrants the belief in drug presence when not a particle of drug matter can be discerned by direct observation, inasmuch as impalpable and invisible material agents, as morbific causes, have often demonstrated their presence by their destructive influence upon the human organism. 8th. That all efforts must fail to attenuate drug matter beyond its ultimate molecule, the division of a molecule being a reduction of the substance into its elements, or the destruction of its identity. 9th. That according to the accepted theory of molecular magnitudes, the ultimate molecule must be reached in the twenty-third decimal attenuation, and that beyond that there must be a gradual diminution in the number of molecules till all are gone. 10th. That neither direct observation, nor analogy, nor anything learned of the conditions and behavior of drug matter, can justify the inference that there is a single molecule of medicine in one grain of the thirtieth attenuation when faithfully made.

Dr. Dake then proceeded to consider the influence of attenuation upon the power of drugs.

In doing so, he noticed some of the leading theories which have been advanced upon the subject; and first, the earliest theory of Hahnemann, and that still entertained by many of his disciples, that drug power may be developed but not increased by the processes of attenuation. That the *potential* medicinal force of a given drug mass is in proportion to the number of its medicinal molecules, and its *actual* medicinal force in proportion to the number of its medicinal molecules made superficial or ready for an immediate contact with nerve tissue, or an immediate absorption and conveyance to its special field in the organism. That attenuation and trituration have for their ends simply the overcoming of cohesion in drug matter and comminution of drug particles.

2d. In later years Hahnemann inculcated not only the development but the great increase of drug power through attenuation. Korsakoff believed in the existence of a drug aura; Lutze believed in animal magnetism being imparted by the hand to the dose employed.

Dr. Bachmann's theory and the recent neuranalytic experiments, and the hypotheses of Dr. Lawton were then considered.

In applying the physiological test to the question under discussion, Dr. Dake referred to Hahnemann's early provings, in which drug power was present beyond any question; to the experiments of Professor Conrad Wesselhœft, those of the Milwaukee Academy of Medicine, and to those of Dr. Sherman and Dr. Potter. From these he concluded that drugs are recognized in attenuations up to the 7th x by their effects upon the healthy human organism, while in the 8th x and 9th x their recognition is less certain.

Dr. Dake concludes his paper with an examination of clinical experience on drug power.

He points out in the first place, the large variety of influence, besides those pertaining to drugs, which may determine recovery. Conversions to high potency views have, he shows, often resulted from a single experience in using them, and this often after a lower attenuation had been in action, though not really fruitlessly for some days. He gives his personal experience on this point, showing that he was nearly led to place confidence in their preparation, because he observed the paroxysms of an intermittent fever suddenly stop after the administration of a single dose of *Arsenic* 200, when he had been exhibiting the 6th and 30th with no apparent benefit. Another case, one of pneumonia, is reported, where, after giving *Bryon.* 3 x with little apparent benefit, a single dose of the 200th was followed by a great change for the better. Reflection, however, convinced him that the change was really due to the preparation which had been previously administered. Dr. Dake further argues, that not one of the cases reported in journals as cured with any high dilutions, furnishes a particle of satisfactory proof that there is medicinal power in attenuations above the thirtieth decimal.

Finally, where homœopathy has gained her greatest victories, as in cholera and yellow fever, the battles have been fought almost entirely by means of the lower attenuations.

### *A Plea for a Standard Limit of Attenuated Doses.*

C. Wesselhœft, M. D., Boston.

Dr. Wesselhœft, after some introductory remarks of a general character on the importance of the question of dose, gives a summary of recent researches that have been made on triturations and dilutions. These point to the fact that the limits of minuteness to which particles of hard insoluble substances can be reduced are arrived at between the $\frac{1}{3740}$th and the $\frac{1}{4527}$th of a millimètre.

Dr. Wesselhœft, in discussing the molecular structure of matter, showed that, whereas in Hahnemann's time it was regarded as infinitely divisible, and that, consequently, homœopathists were on this basis right in proceeding to attenuations, however high, it had now been demonstrated that there was a limit beyond which molecular divisibility did not extend. He then proceeded to estimate, from the calculations and experiments of Sir William Thompson and Professor Clerk-Maxwell, that, with the eleventh centesimal dilution, the number of molecules in a drop of liquid is exhausted. By a series of further calculations, he concludes that the supposition of transmission of molecular force, separated from the original medicine molecules, is untenable in the light of modern molecular science.

Dr. Wesselhœft then argued that the molecular constitution of matter demanded the omission from our statistics of all clinical results obtained with dilutions above the eleventh centesimal. With regard to the value of clinical experience in enabling us to estimate the best standard of dose, Dr. Wesselhœft contended that it is at present but slight, owing to the inadequacy of statistical materials. What is deemed clinical experience consists, he says, of recorded cures, with the entire omission of opposite or negative results, which must be presumed to be large, and a decision will, therefore, be impossible until "experience" includes numerous and accurate statistics of negative as well as of positive results. Dr. Wesselhœft concludes by urging the limitation of the dose to attenuations below the eleventh centesimal.*

---

* *The Homœopathic World* says of this discussion on the posological question:

"Speaking generally, the essays were all against infinitesimals, though no points were really made against them; and a perusal of the essays shows that the various essayists merely go over very old ground, threshing empty straw by the way. Although the essays were *against* the infinitesimals, it soon became

### *The Question of Doses: Hahnemannism and Homœopathy.*

Dr. Cretin, Paris, France.

Dr. Cretin opens his paper by asserting the therapeutic power of infinitesimal doses, but he demands that their degree of this power be ascertained by experiment alone.

He desires to inquire, 1st, What, for each drug, are the limits of its therapeutic action; at what stronger dose does its action commence; at what weaker dose, what attenuation does it cease? These limits being fixed, what is, in each case, the dose which shows itself the most efficacious, the strong, weak, or even the infinitesimal?

Dr. Cretin denies that there is any evidence of Hahnemann's having been led to the use of attenuation in consequence of aggravation from larger doses, but that he proceeded to them by analogies, by indication, by anticipating generalization, and also by studies. This he endeavors to make good by analyzing Hahnemann's pathological illustrations of the law of similars in the *Organon.*

In the following two chapters he examines attenuations, dynamizations, and medicinal aggravations, and then the practice of Hahnemann. From this inquiry he concludes that Hahnemann has not established on any data, rational or experimental, either the necessity, the utility, or the action of the infinitesimal attenuations, and still less the aggravations, which, according to him,

---

evident that the great majority of those present have unabated confidence in them. Following in the wake of the opener, Dr. Burnett, of London, pointed out that the evidence in their favor was overwhelming, for a majority of the very best homœopathic physicians, from Hahnemann down, had lived and died in the firmest faith in the great efficacy of infinitesimals. Dr. Burnett thought Dr. C. Wesselhœft's position had been shown to be untenable by Dr. Buchman, in his essay presented to the Convention by the Homœopathischer Central-Verein of Germany. He called attention, moreover, to the remarkable fact that almost all the older opponents of infinitesimals *were themselves brought over to homœopathy by observing the effects of these same infinitesimals; and also that these self-same gentlemen, who now seek to ridicule the infinitesimal dose, scored their own greatest successes at a time when they used infinitesimals, almost exclusively,* in their practices. Dr. Burnett did not advocate the exclusive use of infinitesimal doses, but put in a plea for the whole range, from the crude drug right up to CM.'s, or higher."

The venerable Dr. Dunn heartily indorsed Dr. Burnett, and called upon the younger men to be faithful to the truth, and not to remove the old landmarks that had been to him a guide through a long and successful professional career.

Dr. Helmuth, New York, made a spirited speech in favor of infinitesimals. Dr. Blackley (author of a work on "Hay Fever"), argued in favor of the efficacy of infinitesimals from his own microscopical observations on certain exceedingly minute bodies. Altogether, the feeling went very strongly in favor of the efficacy of the infinitesimals.—*Homœopathic World.*

should be at once the proof of the condition and the product of their action.

The clinical aspect of the infinitesimal dose shows that the admission of its power rests upon an experimental basis. The questions then arise, at what dose does medicinal action begin—at what attenuation does it cease? And again, are infinitesimal doses preferable to appreciable doses in all cases, or in what cases only? A lengthened inquiry in using all dilutions from the 30th downwards has, Dr. Cretin says, convinced him that the action of a drug is less sure as the attenuation is high. "In acute, as in chronic affections," he adds, "I have never obtained from the higher dilutions the results which have been given me in a more positive fashion by the dilution below the sixth, and, above all, by the unattenuated medicine."

With some remarks on the choice of the dose in individual medicines, and a comparative view of Hahnemannism and homœopathy, Dr. Cretin brings his essay to a close.

A discussion followed on the relative value of clinical and extra clinical evidence as to the efficacy of the infinitesimal dose.

On the following morning (Thursday) a sectional meeting was held, of members especially interested in gynæcological studies. The chair was taken by Dr. Eaton, of Cincinnati. The papers on this subject to be brought forward in the afternoon formed the basis of discussion.

In the afternoon, at the general meeting, business commenced by the presentation of papers, of which the following are abstracts:

### *On the Differential Diagnosis and Treatment of Yellow Fever.*

WM. H. HOLCOMBE, M. D., New Orleans.

After a full definition of yellow fever, Dr. Holcombe spoke of its geographical range. It is endemic in the islands and cities of the Atlantic coast of tropical America. From this habitat it may be transported northward and southward many degress of latitude, but very few of longitude. Yellow fever has no second week. It and plague are the shortest of all febrile diseases, as they are also the most fatal. Yellow fever becomes more fatal as it advances northward. It is the hottest of all fevers. It is a hæmorrhagic fever, the hæmorrhages depending on chemical changes in the blood itself. The jaundiced or icteric condition is a peculiarity of the fever, and is entirely of blood origin. An abnormally slow pulse down to 50, 40, and even 30 pulsations is found in many cases. Yellow fever has a melancholy pre-eminence in its marked or latent features, its sudden changes and terrible surprises requiring more watchful care and vigilant nursing than any other disease, the danger being often out of proportion to the symptoms.

Dr. Holcombe then described the *post-mortem* appearances of yellow fever, and then proceeded to compare its phenomena with those of the other great fevers. In speaking of the treatment of yellow fever, Dr. Holcombe laid especial stress on the importance of nursing and hygiene—a sudden noise, movement in bed, conversation, a piece of bad news, any excitement, the presence of food in the stomach at the wrong time, the omission of a stimulant at the right moment, being often enough to transform a hopeful into a hopeless case.

Of the medicinal treatment, Dr. Holcombe says that we have no specific for the first or febrile stage of yellow fever. His paper concluded as follows:

"It is in the second stage of fever, when we contend with local congestions, special inflammations, and the effects of blood poisonings or other morbid processes, that homœopathy asserts its specific and unquestionable power. We may not be able to break or materially shorten the continued fevers, but we can control the bronchitis of measles, the sore throat of scarlatina, the suppuration of smallpox, the pneumonia of typhus, the diarrhœa of typhoid, the jaundice and hæmorrhages of yellow fever, etc., in the most remarkable manner, thereby reducing the mortality of all those diseases to a point considerably below the acknowledged allopathic level.

"What enormous services have been rendered in these cases by those chemically isomorphous substances, *Arsenic*, *Phosphorus* and *Tartar Emetic*, applied upon the homœopathic principle! To these may be added, as special remedies for yellow fever, the snake poisons, *Lachesis*, *Crotalus*, *Naja Tripudians*, *Elaps Corallinus* and *Vipera Torva*, introduced into practice from the long-recognized resemblance between the symptoms of yellow fever and those which have followed the bite of serpents. These serpent poisons will no doubt be found valuable also in the hæmorrhages and jaundice of the plague, of typhus, relapsing fever, bilious typhoid and malignant remittents.

"The homœopathic treatment of yellow fever is still in its infancy, comparatively speaking, but the results already achieved constitute one of the strongest arguments ever offered in behalf of the practice."

### *Indian Dysentery and Cholera.*

P. W. Carter, Ph.D., L.M., etc., Sydney.

This paper opens with a minute account of the phenomena of Indian dysentery. Then follow a series of well-reported cases of the disease. Dr. Carter makes the following statement of the results of his practice while in India: "The total number of cases," he says, "treated by me allopathically up to the November, 1875, was 213—deaths 99. Cases treated homœopathically up to the end of 1878 (I left India in March, 1879) were 77, with 14 deaths—all in dispensary practice, when the disease, and every disease, is generally seen in an advanced stage."

With regard to cholera, Dr. Carter had seen little advantage from the use of *Camphor* even in the stage of invasion. In the first stage, he says, he did best with *Aconite* 1x or *Φ*. This, when given early, prevented the advancement to the second stage in every instance. In the second stage, *Verat. alb.* 3x, *Arsen.* 3, *Cup. acet.* 2 or 3, *Sec. cor.* 3x, *Ant. Tart.* 3x and 3, and *Croton* 3 were the chief and most reliable remedies. In the stage of collapse, *Arsen.* 30 was used with the happiest results. In pulmonary congestion, *Phos.* 3 or 5. When this had grown to blood-poisoning, with brain symptoms, *Bell.*, *Stram.*, *Hyosc.* or *Ac. Hydrocy*, were used with better effect than any treatment he had obtained under old-school practice. Three out of four cases of intra-cranial effusion yielded to *Digitalis.* In renal congestion, with albuminuria or suppression and uræmia, he found *Terebinth.* 3x, *Kali bich.* 3, *Canth.* 2 or 3, and *Digit.* 3x very effective.

---

### *Homœopathy in the Treatment of Diseases prevalent in India.*

Mahendra Lal Sircar, M.D.

The paper sent in by Dr. Sircar was found too lengthy for the *Transactions*, and to cover more ground than had been intended. Such portions only were introduced to the Convention as bore upon the therapeutics of the special types of Indian disease.

Diarrhœa, generally traceable to bad food, but sometimes to extremes of temperature, was first noticed, and the indications given for the use of *China, Arsen*, *Coloc.*, *Puls.*, etc. Of dysentery, Dr.

Sircar says: "In the majority of cases I find *Ipecac.* to be quite competent to deal with the disease. Failing this, I have recourse to the *Merc. sol.*, and in very grave cases to *Merc. cor.* Other medicines meeting special cases, are *Aconite*, *Bellad.*, *Canth.*, *Capsicum* and *Colchicum.*"

The liver is an organ very frequently disordered in India. In malarious enlargement, remedies that are suitable for the general condition, prove corrective of it. *Acon.* and *Bry.* in febrile states; *Calc. c.* especially in young children; *Nux. v.* when there is constipation; *Lycopod.* when with constipation there is tympanitis, especially of the colon. In acute congestion, no remedy equals *Aconite;* sometimes *Bryonia* is required subsequently. When the secretory structures are inflamed, *Mercury* is wanted. In suppuration, *Aconite* and then *Cinchona* or *quinine* in massive doses. In very prostrate conditions, *Arsenic*, *Carb. v.* and *Lachesis.*

In hypertrophic cirrhosis with jaundice, *Lachesis* is a capital remedy. In chyluria, Dr. Sircar has seen good done by *Carb. v.* and *Phosph. acid.* In hydrocele and elephantiasis of the scrotum, Dr. Sircar has seen benefit derived from *Silica*, *Rhododendron*, and sometimes from *Rhus.*

---

### *Malarious Fever in India.*

PRATAP CHANDON MAJUMBA, L. M. S., etc., Calcutta.

This communication was one of inquiry rather than one presenting good therapeutic results. Dr. Majumba says that *quinine*, which is almost the only drug resorted to, does more harm than good in many cases—though useful in some. So far as his experience has gone, he has found *Aconite* useless. *Bell.*, in some cases of a remittent type, has proved serviceable; so, also, has *Gelseminum*, especially in children with a delicate nervous system. *Baptisia* followed by *Bryonia*, *Rhus*, *Arsenic* and *Muriatic acid*, have been of great value in cases where the fever has assumed a typhoid type. Dr. Majumba concludes by remarking on the necessity of a careful study of the materia medica in each case, etc.

These papers having been introduced by the President, a discussion followed on Homœopathy in Hyper-acute Disease, including Hyper-pyrexia.

The subject of *Cancer* was then brought before the Convention in a paper by Dr. Gutteridge, of which the following is an abstract:

After some reference to the statistics of cancer, and having given a definition of the disease, Dr. Gutteridge expressed his doubts as to the value of microscopic observation and chemical analysis as means of diagnosis. Referring to the researches of Haviland on the geographical distribution of disease, he showed that districts where the mortality from cancer was high were such as are liable to somewhat long-continued floods from the overflowing of rivers. He then entered on a somewhat minute differentiation of cancer and simple glandular enlargement. Passing to the consideration of the propriety of operation, he showed that extirpation by the knife does not cure cancer, does not always remove it, and that the liability to return is ever present, and often an absolute certainty. The results of enucleation, he says, are in no way more favorable. He concludes, therefore, that cancer patients do better when treated medicinally alone. In scirrhus he pointed out the indications for *Bell.* and *Conium.* *Cicuta* is also named as useful. Of all most generally useful remedies, Dr. Gutteridge speaks most favorably of *Hydrastis*, and especially of Tilden's preparation, *Hydrastin*, intimately incorporated with an equal quantity of *Hydrastis.* When this drug is given internally, a lotion of the tincture or powdered root should be applied at the same time. When ulceration has taken place, Dr. Gutteridge laid great stress on the value of *Hydrastis*, *Hamamelis*, *Comocladia*, *Baptisin*, and the *Iodide of Arsenic*, pointing out the special indications for the use of each.

In epithelioma, Dr. Gutteridge drew attention to *Ranunculus*, *Arsenic* and *Hydrastis* as medicines from which the best results had accrued. In discussing the treatment of cancer of the stomach, he pointed out the indications for the use of *Ranunculus*, *Phosph.*, *Argent. Nitric.*, *Arsenic*, *Hydrastis* and *Baptisia.* With some observations on the nature of the diet best adapted to cases of cancer, Dr. Gutteridge concluded his paper.

A discussion ensued on the Possibilities of Medicine in Cancer.

Papers were then presented on gynæcological subjects, the first being by Dr. Edward Blake, *On the Place of Mechanical Measures in Pelvic Disease.*

After some introductory remarks on the anatomy and physiology of the uterus, Dr. Blake argued that the greater number of the disorders of the female pelvis may be included in four categories—1, Mechanical changes acting from without; 2, Mechanical changes acting from within; 3, Physiological changes acting from without; 4, Physiological changes acting from within.

"The inclination," said Dr. Blake, "of the dominant school of therapeutics, is probably whilst attaching undue importance to mechanical methods to ignore the second or vital side; whereas our own tendency as undoubtedly is to decry the former."

Dr. Blake said that during the first six years of his practice he abjured local physical examination almost entirely, and worked laboriously at subjective symptomatology, with comparatively unsatisfactory results; that during the succeeding six years he turned his attention to the use of various means of physical diagnosis, but without using any mechanical contrivances for the purpose of local treatment; while during this time he frequently witnessed through homœopathy the temporary removal of results of morbid processes without necessarily attacking the cause; he never during this time witnessed the smallest cervical excoriation healed under the influence of internal medication alone, even when such medication was carried on under the most favorable circumstances. Subjective symptoms Dr. Blake relies on to differentiate between a group of closely allied remedies, but to lead up to that group for diagnostic and prognostic purposes he trusted solely to objective signs.

Dr. Blake concluded his paper by urging greater attention to the mechanical causes of diseases.

---

### *On the Treatment of Common Metritis, especially that Form known as Endo-Cervicitis, with Ulceration of the Cervix.*

D. Dyce Brown, M. A., M. D.

Dr. Brown commenced his paper by dwelling on the imperfections which exist in our provings, so far as they relate to chronic uterine inflammation. A medicine to be selected in this class of disease must show—1st, from the provings filled up by the results of clinical observation, that it has a specific relation to the genital organs by producing disorded menstruation, leucorrhœa, ovarian pain, etc.; or, 2d, if the symptoms should be scanty in the provings, the medicine must be one which shows a specific affinity for mucous membrane in general, producing catarrh or acute inflammation, with their results in the shape of increased secretion or ulceration; or, 3d, it is of the utmost importance that it should "cover" the constitutional dyscrasia that may be present with the various symptoms referable to other organs than the uterus and ovaries. In other words, it must cover the totality of the symptoms.

The greatest amount of success Dr. Brown thought was attainable, when a remedy is selected which covers the general state of disordered health, more especially if this remedy is known to have a specific affinity for the uterine organs.

Before considering medicines, Dr. Brown drew attention to local applications. Weak solutions of astringent remedies he regarded as acting in accordance with the homœopathic law in cases of chronic inflammation. When first practicing homœopathy, he thought that such applications as *nitrate of silver* hastened the cure of disease of the cervix. Clinical observation had, however, convinced him that with specific general treatment such applications as *nitrate of silver*, *Iodine*, *Carbolic acid*, applied by the mop through the speculum were unnecessary. Just, however, as every one would use water dressing or *Calendula* or *Hydrastis* to promote healing in ulcerated surfaces, so he employed these means in such cases. When in addition to ulceration the cervix was hypertrophied, *glycerine* diluted with water or with a few drops of *Hydrastis* added, was useful. Where vaginal catarrh is excessive injection of *Calendula* and *Hydrastis*, or *even in chronic cases of a weak solution of zinc or alum, were beneficial.* In suitable cases, Dr. Brown attached great importance to the wet compress and to the tepid sitz bath.

Dr. Brown then pointed out the indications for the use of medicines. *Belladonna*, he said, was required in almost every case of chronic cervicitis with ulceration at some period of its progress. The indications were fully and minutely given, but at too much length to allow of our transcribing them here. *Sulphur* he found often required, especially in cases of chronic inflammation of the venous type—when that sluggish state of the system exists which refuses to respond to the action of medicines. The symtomatological indications were then given. *Sepia*, he showed, was indicated in endo-cervicitis, where the uterus is enlarged, prolapsed, or where version has occurred. When there is a tendency to skin eruptions, etc., *Pulsatilla*, he pointed out at some length, was indicated in cervical disease by the appearance, complexion, and temperament of the patient, the scanty or irregular menstruation, the menstrual pain, the leucorrhœa, prevailing chillness, aggravation of symptoms in the evening, but especially by the gastric or gastro-intestinal catarrh with headache. *Actæa* corresponded to the nervous, neuralgic, hyper-æsthetic patient suffering from uterine disease. The coincidence of cervical inflammation, slight or severe, with well-marked hyper-æsthesia (showing itself by the spinal tenderness, the peculiar head-aches, the palpitation and sleeplessness from mental depression, or alternation of depression with excitement, and sinking pain at the epigastrium) indicates the kind of case in which it is useful. *Ignatia* was indicated rather by the general state of nervousness that characterized some cases than by local manifestations of disease. *Calcarea carbonica* in cervical disease associated with struma he describes as a remedy of immense value, especially if the catamenia are too frequent and profuse. *Lycopodium* is useful in cases where the pelvic organs are congested and leucorrhœa and endo-cervicitis are set up in consequence of the liver and portal circulation becoming congested. The condition requiring *Nux vomica* resembles that in which *Lycopodium* is useful. *Mercury* is especially indicated in cases of endo-cervicitis, when the ulceration is of an unhealthy and sloughy type, and when vaginal catarrh with thick leucorrhœa is present to a marked degree; 2, when gonorrhœa has extended upwards to the uterus; 3, when syphilitic ulceration is made out, or when there is reason to expect a syphilitic taint; 4, when the collateral symptoms, those of the stomach, liver, and intestines, especially call for *Mercury*. Dr. Brown also noticed *Lilium*, *Graphites*, *Arsenic*, and *Platina* as often indicated in uterine disease, and concluded by saying that, in his opinion, we quite as often require to select our remedy less on the grounds of its local action than on those of the systemic disturbance or constitutional taint which may be present in a given case, and the more carefully such selection is made, the better it seemed to him were the results.

---

*On the Treatment of some of the Affections of the Cervix Uteri.*

GEO. M. CARFRAE, M. D.

Dr. Carfrae commenced with some remarks on the unsatisfactory character of much of the materia medica, and this especially as related to the action of medicines on the cervix uteri. Restricting his attention to the consideration of cervical endo-metritis, or cervical catarrh, or uterine leucorrhœa and granular erosion, or ulceration of the cervix, he entered into a full account of the etiology, symptomatalogy, and pathology of the condition. Passing to the treatment, he divided it into constitutional and local. In discussing the former, he took Guernsey's book on *Obstetrics*, and examined the medicines named therein as applicable to this condition. He insisted that as leucorrhœa was a constant symptom of this disease, it ought to be among the phenomena produced by each medicine adapted to cure it, if the totality of the symptoms was to be our guide. Many of the medicines recommended by Guernsey have not this symptom in their provings. Of the provings of others, it must, he thought, be admitted that they were unreliable. He then proceeds to examine *seriatim* all the medicines named by Guernsey, concluding that of out seventy-two such remedies, about a dozen and a half have no leucorrhœa in the list of symptoms attributed to them; while about one-half of the whole number have been proved, Dr. Carfrae thinks, in a manner too loose to merit our confidence, reducing the number of drugs, the provings of which entitle us to look upon them as truly homœopathic to cervical leucorrhœa to scarcely a dozen: and of these Dr. Carfrae is doubtful of at least six. Of eleven other medicines recommended by Hale, the value is chiefly empirical, few of them having been thoroughly proved.

Regarding the materia medica as poor in relation to truly homœopathic remedies in cervical leucorrhœa and granular and follicular disease of the cervix, he asks, do we get any help from local applications, and if so, from what? He then examines the views of Guernsey, Madden, Leadam, Ludlam and Hale, with regard to the use and mode of action of externally-applied irritants. He concludes that we are far from having arrived at that amount of scientific precision which is desirable or attainable. This he attributes to some extent to the number of unreliable provings which are incorporated in our text-books. To some extent, also, it is due to the difficulty of getting good provings of drugs which have a specific relation to the uterus; while, lastly, the semeiology of these affections is often very vague, and no sure indication of their pathological condition. To admit that the combined local and constitutional treatment of cervicitis, granular, erosion, etc., gives the patient the best hope of a cure, is to allow that our treatment is to a certain extent empirical. "This," he adds, "I fear must be so, until we have a reformed materia medica." As medicines, Dr. Carfrae relies chiefly on *Arsenic*, *Mercurius*, *Nux Vomica*, *Phosphorus*, *Pulsatilla*, *Sabina*, *Sepia*, and *Ferrum*, while *Gelseminum*, *Helonias*, *Hamamelis*, *Lilium*, *Phytolacca* and *Xanthoxylum* are, he thinks, valuable additions to our armamentarium, but requiring more thorough proving. The best local applications are *chromic*, *carbolic*, and *nitric acids* and *nitrate of silver*.

He concludes by hoping that ultimately we may treat these cases altogether without the aid of local applications. So long as these are used, we must admit that our treatment is, to a certain extent, unscientific and unsatisfactory. When we can abolish them, it will be because we have attained that amount of scientific precision which meanwhile it must be our constant endeavor to reach.

A discussion followed on the Influence of Homœopathy on Uterine Disease, at the conclusion of which the meeting adjourned.

On Friday afternoon the subject of general, ophthalmic, and aural surgery were brought under the consideration of the Convention, and received full discussion.

The first contribution presented was from Dr. Bojanus, of Nischny-Novogorod, in Russia. It was in the form of a book, entitled *Homœopathic Therapeutics in its application to Operative Surgery;* and upon this Dr. Dudgeon prepared a report, giving a brief *resumé* of its contents. It is occupied with a detailed analysis of the operations performed in the hospital to which the author is attached.

*Surgical Therapeutics* is the subject of Dr. J. C. Morgan's (Philadelphia) contribution to the *Transactions.*

Dr. Morgan commences his paper with some remarks on the comparative value of *Aconite* in wounds and other injuries. In these classes of cases, Dr. Morgan contends that *Aconite* is superior to *Arnica*—1, in injuries of the eyeball; 2, in the reaction which occurs some hours after an injury; 3, in the commencement of a sprain. Dr. Morgan then adduces some illustrations of the sorbefacient effects of the internal exhibition of *Hydratis* 30, *Sepia* 1m, *Arsen. iod.* 3x and *Hypericum* 2x in mammary tumors.

Passing to tumors of the uterus and ovaries, Dr. Morgan has no records of absolute cure by drugs, but he can say that in no case has it it been necessary to submit any such to a surgical procedure, except the pedunculated polypi, fibrous and mucus; these he has uniformly removed by the wire *écraseur.* All others he has treated with drugs "in potency" for months and years, according to the various changes of symptoms, to the great satisfaction of patients, who, in sheer desperation, had previously courted the most formidable resources of surgery.

Dr. Morgan concludes by giving the characteristic indications for the use of a number of medicines in the treatment of tumors.

Dr. Watson, of Hammersmith, contributed a paper entitled *Surgical Observations,* which consisted of some general observations on the pathology and treatment of abscess, illustrated by several cases.

A discussion then ensued on the Help brought to the Surgeon by Homœopathy, in which Dr. Dunn, Dr. M'Clelland, Dr. Helmuth, and others took part.

A Paper on the *Therapeutics of Iritis*, by Dr. Vilas, of Chicago, was then presented.

Dr. Vilas declined to discuss the curability of iritis by internal remedies alone, because he is of opinion that internal medication alone will never cure all cases which might be cured were they treated with all the means at our command. The first point in the treatment, he says, consists in perfect rest of both eyes, shutting out of bright light, and protection from injurious changes of temperature. The second consists in obtaining complete rest for the iris. Of all mydriatics, *atropia*, he said, was the best, and the best preparation a carefully prepared *sulphate*. The advantages to be obtained and dangers to be avoided were fully pointed out. Various other mydriatics were noticed by Dr. Vilas. In all cases, save those in which there are no synechiæ likely to form, can, he alleged, a mydriatic be safely dispensed with. If there be exudation from the iris, and it is not drawn away from its resting-place, synechiæ must form, and more or less firmly tie down the iris. Dr. Vilas next considered the indications for the use of internal remedies. These comprised some twenty-eight drugs, and form a useful collection of references for ophthalmic surgery. We must, however, direct our readers to the *Transactions* for their study.

The Treatment of Iritis, simple and syphilitic, was then the subject of discussion, the debate being opened by Dr. Bushrod James, of Philadelphia.

This being terminated, the last paper to be presented to the Convention, that by Dr. Cooper, of London, on Aural Surgery, was introduced under the title, *Notes on some Homœopathic Remedies in Aural Disease.* After some introductory remarks on the position of the therapeutics of aural surgery, Dr. Cooper pointed out the indications for the use of the following medicines in different forms of deafness: *Gelseminum, Hydrastis, Canadensis, Picric acid, Capsicum, Arnica, Rhus, Ignatia, Quinine, Amyl nitrite, Chloroform, Salicylic acid* and *Salicylate of soda, Apis mellifica, Lachesis, Elaps cor., Crotalus, Formica, Naja* and *Vespa.* In reviewing his experience, Dr. Cooper says that the conclusion is forced upon him that very long standing cases are best met by highly dynamized preparations; these, beyond question, he says, exert a most powerful and satisfactory influence. He especially names *Phos.* and *Calcarea* as remedies which in a high dilution have proved of most essential service.

After Dr. Cooper's paper had been introduced, a discussion ensued on the plan of Homœopathic Medication in Ear Disease.

The Convention assembled at two o'clock on the following day for the transaction of miscellaneous business.

The report of the Committee and the President's address were brought forward, and as practical results it was determined to appoint a committee, consisting of one or more skilled pharmaceutists in each country represented by the Convention, to co-operate with the editor of the *Pharmacopœia of the British Homœopathic Society* in the preparation of a pharmacopœia which shall be adopted by all nations.

It was also resolved that a permanent secretary of International Homœopathic Conventions be appointed, and to this office Dr. Richard Hughes was unanimously appointed.

After some conversation, it appeared to be the wish of the members of the Convention that the meeting of the Convention, which would in the ordinary course of events be held in 1886, should take place at Brussels.

The statistics of the Convention were presented by the President, from which it appeared that 78 British, 31 American, 4 French, 1 Italian and 1 Russsian physician had entered their names on the books of the Congress, while there is reason to believe that some 20 British practitioners had been present at the meetings, but had omitted to record the fact of their presence.

After very cordial votes of thanks to the President, Vice-President, Secretaries and Treasurer, the members separated.

---

We have devoted considerable space to this Convention of *physicians practicing homœopathy.* Our readers will see that there is very little true homœopathy in these discussions. The time seems to have been occupied chiefly in abusing Hahnemann and attempting to show what homœopathy *is not and what it cannot do*—in their hands. As these physicians *practicing homœopathy* have never prescribed homœopathically, and as they, for the most part, know nothing of the homœopathy of Hahnemann, the only result they could expect from their practice was failure. This they acknowledge, for the most part, they have achieved. The proceedings of this Convention will serve to furnish another Dr. Smythe with arguments against homœopathy. It also serves to show the great necessity for the *International Hahnemannian Association.*

